# Off-Road Vehicle Crash Risk during the Six Months after a Birthday

**DOI:** 10.1371/journal.pone.0149536

**Published:** 2016-10-03

**Authors:** Jason D. Woodfine, Deva Thiruchelvam, Donald A. Redelmeier

**Affiliations:** 1 Department of Medicine, University of Toronto, Toronto, Canada; 2 Evaluative Clinical Sciences Program, Sunnybrook Research Institute, Toronto, Canada; 3 Institute for Clinical Evaluative Sciences in Ontario, Toronto, Canada; 4 Division of General Internal Medicine, Sunnybrook Health Sciences Centre, Toronto, Canada; 5 Center for Leading Injury Prevention Practice Education & Research, Toronto, Canada; University of New South Wales, AUSTRALIA

## Abstract

**Background:**

Off-road vehicles are popular and thrilling for youth outside urban settings, yet sometimes result in a serious crash that requires emergency medical care. The relation between birthdays and the subsequent risk of an off-road vehicle crash is unknown.

**Methods:**

We conducted a population-based before-and-after longitudinal analysis of youth who received emergency medical care in Ontario, Canada, due to an off-road vehicle crash between April 1, 2002, and March 31, 2014. We identified youth injured in an off-road vehicle crash through population-based health-care databases of individuals treated for medical emergencies. We included youth aged 19 years or younger, distinguishing juniors (age ≤ 15 years) from juveniles (age ≥ 16 years).

**Results:**

A total 32,777 youths accounted for 35,202 emergencies due to off-road vehicle crashes within six months of their nearest birthday. Comparing the six months following a birthday to the six months prior to a birthday, crashes increased by about 2.7 events per 1000 juniors (18.3 vs 21.0, p < 0.0001). The difference equaled a 15% increase in relative risk (95% confidence interval 12 to 18). The increase extended for months following a birthday, was not observed for traffic crashes due to on-road vehicles, and was partially explained by a lack of helmet wearing. As expected, off-road crash risks did not change significantly following a birthday among juveniles (19.2 vs 19.8, p = 0.61).

**Conclusions:**

Off-road vehicle crashes leading to emergency medical care increase following a birthday in youth below age 16 years. An awareness of this association might inform public health messages, gift-giving practices, age-related parental permissions, and prevention by primary care physicians.

## Introduction

Off-road vehicles are a popular means of transportation [1], recreation, and industry away from urban regions worldwide [2–3]. Modern definitions include all motorized vehicles used on rough terrain and include two-wheeled dirt-bikes, three or four wheeled all-terrain vehicles, and snowmobiles [4]. Unsafe riding is problematic, however, due to limited rider protection [5], lack of safety restraints [6], difficult trail terrain [7], exposure to adverse weather, high impact velocities [8], and remote crash locations [9–10]. Most regions, therefore, have regulations against alcohol intoxication [11], riding with excess passengers, or failure to wear a helmet [12–13]. Yet risky behavior persists due to popular attitudes among riders [14] and impractical enforcement strategies for off-road vehicles [15–16].

Unintentional injury is a leading cause of morbidity and mortality in youth [17–20]. The general risk of unintentional injury is partially caused by a propensity for risk-seeking [21], impulsive behavior [22–24], peer pressure, and limited judgment [25]. The risk might be further accentuated by a lack of positive role modeling [26–27], inadequate supervision [28], defiant personal mindsets [29], and recreational drug usage [30]. The popular media sometimes also encourages unsafe behaviors, as with the reporting of numerous celebrities influencing youth toward joy riding [31–36]. Both the incidence and severity of off-road vehicle crashes during modern years are increasingly skewed toward young riders in rural, remote, underserviced, and other marginalized settings [37–40].

The literature provides no information on how a birthday might influence the subsequent risk of an off-road vehicle crash among youth. Birthdays are among the most noteworthy landmarks of childhood and are associated with distinct changes in status, privilege, identity, and responsibility [41–43]. Parents, for example, may enable usage by connecting the freedom to ride with a child’s specific birthday. On the other hand, a youth may also experience increased freedoms of all sorts that obscure the effect of any single lifestyle risk [44–45]. We conducted a before-and-after longitudinal study using linked health databases to test whether birthdays were associated with an increased subsequent risk of a serious off-road vehicle crash among youth. We tested the null hypothesis that the risk of a crash would be the same in the 6 months following a birthday compared to the 6 months preceding a birthday.

## Materials and Methods

### Study Setting

Ontario is Canada’s most populous region with a youth population (age < 20) of 3,117,893 in 2008 (study midpoint) [46]. The population was dispersed over a land area of 917,741 square kilometers (larger than the entire American Midwest), yielding a total population density of 14.1 per square kilometer (similar to Kansas) [47]. A total of 639,834 off-road vehicles were registered in Ontario in 2008 [48] [49]. Unlike bicycles, off-road vehicles required government-approved plates, insurance coverage, and formal registration with the Ministry of Transportation. Although Ontario law mandated that riders wear helmets (a legal standard still in effect at the time this manuscript was written) and limit alcohol consumption [50] youth of any age were permitted to operate a vehicle with no licensing examination required [51]. During the entire study, Ontario health insurance covered emergency department care throughout the region with no out-of-pocket costs to patients. Our study protocol was approved by the Sunnybrook Research Ethics Board and included a waiver of individual consent. Our analysis used individual patient data housed at the Institute for Clinical Evaluative Sciences in Ontario and data access is available online at the ICES Data Access webpage.

### Identification of Youth

We identified youth injured in an off-road vehicle crash through established population-based health-care databases of individuals treated for medical emergencies throughout Ontario from April 1, 2002 through March 31, 2014 (reflecting all data available) [52–55]. Patients below age 20 years (age < 20 years) were defined as youth, and eligible for inclusion. Youth below age 16 years (age ≤ 15 years) were defined as juniors, in accordance with prevailing legal restrictions against on-road driving [56]. The remaining youth above age 16 years (age ≥ 16 years) were defined as juveniles, in accordance with the legal limit for initiating on-road driving. Youth with missing health card numbers, faulty records, blank identifiers, or declared dead at the scene as coroner’s cases were not included due to unavailable data.

### Defining a Birthday

Each youth’s birthday served as the primary predictor under the assumption that such milestone events might be linked to lifestyle changes. For analysis we identified the nearest birthday relative to the crash date for each individual. For example, a youth born on August 1, 2001 and injured on August 7, 2012 was coded as having a crash 6 days after their birthday. Conversely, a youth who had the same crash date but was born on August 27, 2001 was coded as having a crash 20 days before their birthday. If multiple crashes related to multiple separate birthdays, we analyzed individuals according to the birthday associated with the earliest identified crash. When necessary, those born on February 29 were assigned a birthday of March 1 to ensure that each youth had exactly one birthday each year.

### Characteristics of Youth

Additional baseline characteristics were defined at the time of the youth’s birthday and obtained through computerized linkages to health care records [57]. The official vital statistics registry served as the source of data on age, sex, and home location (urban or rural) [58]. The Statistics Canada algorithm based on neighborhood income quintile was used to estimate socioeconomic status, as validated in past research [59–61]. The physician services database provided data on prior hospitalizations, emergency visits, and outpatient contacts in the prior year [62–65]. The available databases contained no information on distances travelled, formal training, community by-laws, peer relationships, adult supervision, personality, or other determinants of behavior.

### Emergency Department Visits

The main study outcome was an off-road vehicle crash that led to an emergency department visit (ICD-10 codes: V20-V29, V30-V39, V86) [4]. In secondary analyses we distinguished dirt-bikes from more stable vehicle types as well as examined the role of birthdays on helmet use, seating position, and night riding. The time lag between the date of each crash and the date of the nearest birthday was calculated based on a pre-specified ascertainment interval of 6 months before and after each birthday to allow sufficient sample size and consistent non-overlapping time spans for all comparisons. The emergency department databases also provided 7 indirect measures of injury severity assessed as ambulance involvement, triage urgency, concussion, transfusion, hospital admission, critical care unit admission, and discharge status.

### Statistical Analysis

The primary analysis examined the incidence of crashes and compared counts before and after each birthday for each youth (self-matched control design) [66]. Our statistical analysis defined the point of reference as the birthday nearest to the crash date so that each youth served as their own control. We characterize the six months preceding a birthday as “before” and the six months following a birthday as “after” in our statistical analysis. For example, an individual born August 21, 1996 and injured on August 1, 2012 was defined as 15 years old at the time of their crash but nearly 16 years old in statistical analysis [67–68]. We analyzed only crashes associated with the first identified birthday, therefore, the counts provided in our figures represent a subset of the total number of crashes that an individual might experience over their lifetime. Graphical displays of incidence were plotted using histograms of equal time segments of 7-day duration. Secondary analyses extended analogous models to examine relevant subgroups. Missing data were coded explicitly as missing where applicable. All p-values were two-tailed and calculated with exact 95% confidence intervals.

## Results

A total of 32,777 youths accounted for 35,202 emergency visits for an off-road vehicle crash within 6 months of their birthday during the study. The majority of individuals (30,577) had only a single observed crash, a few (2,004) had exactly two crashes, a few (168) had exactly 3 crashes, and a few (28) had more than 4 crashes. The modal junior was a 12-year-old boy with higher socio-economic status and almost half lived in a rural location (Table 1). The modal juvenile was 17 years old; otherwise, the distribution of baseline characteristics were similar for juniors and juveniles at the time of the crash. A high proportion of emergencies occurred in spring or summer, during the afternoon, and on weekends. About one-in-thirty had a past history of concussion, whereas about one-in-seven had a past history of a disruptive behavioral disorder (S1 Table).

**Table 1:** Characteristics of Patients and Crashes.

Patient Characteristic	Juniors (n = 20,359)*	Juveniles (n = 12,418) †
**Sex**		
Boys	15,316 (75%)	9,518 (77%)
Girls	5,043 (25%)	2,900 (23%)
**Socio-economic status**		
Highest	4,226 (21%)	2,504 (20%)
Next to highest	4,609 (23%)	2,821 (23%)
Middle	4,355 (21%)	2,572 (21%)
Next to lowest	3,860 (19%)	2,433 (20%)
Lowest	3,067 (15%)	1,973 (16%)
Missing	242 (1%)	115 (1%)
**Home location**		
Urban	12,032 (59%)	7,357 (59%)
Rural	8,322 (41%)	5,054 (41%)
Missing	≤5 (0%)	7 (0%)
**Prior hospitalizations §**		
≥ 1	388 (2%)	357 (3%)
**Prior emergency visits §**		
≥ 1	7,766 (38%)	5,418 (44%)
**Prior outpatient contacts §**		
≥ 7	3,083 (15%)	2,226 (18%)
Crash Characteristic	Juniors (n = 21,693) *	Juveniles (n = 13,509) †
**Day of week**		
Weekday	11,964 (55%)	8,211 (61%)
Weekend	9,729 (45%)	5,298 (39%)
**Time of day**		
Morning	3,179 (15%)	2,083 (15%)
Afternoon	13,629 (63%)	7,661 (57%)
Night	4,885 (23%)	3,765 (28%)
**Helmet use**		
Yes	6,143 (28%)	3,719 (28%)
No	15,550 (72%)	9,790 (73%)
**Position**		
Driver	12,988 (60%)	8,828 (65%)
Other	8,705 (40%)	4,681 (35%)

**Fig 1:**
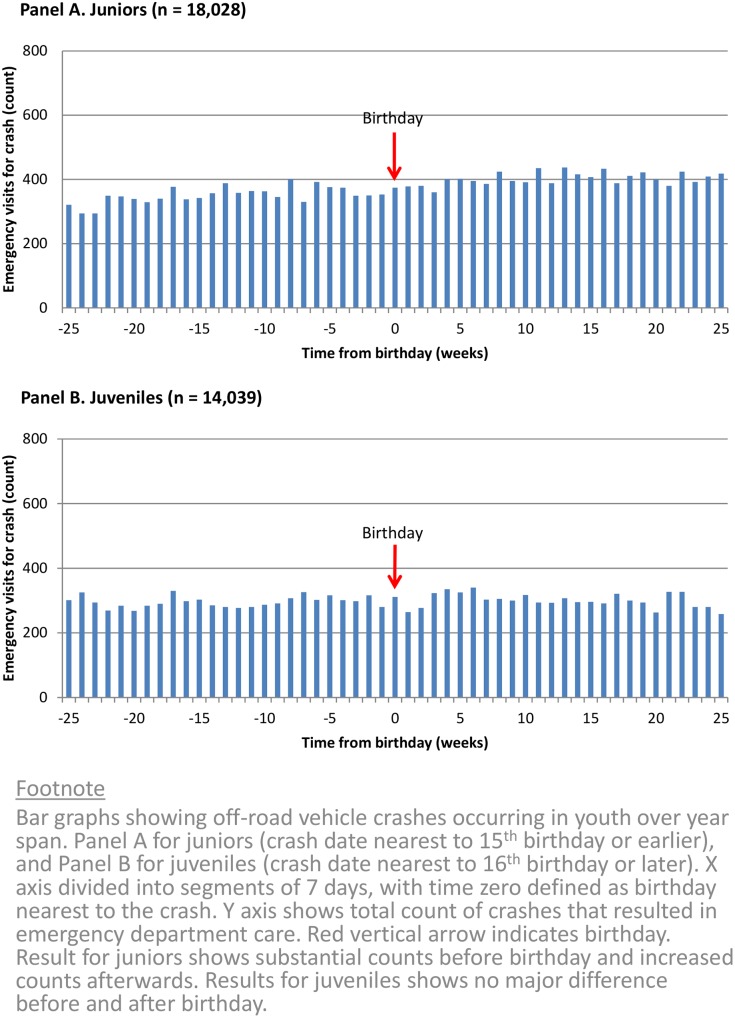
Emergencies from off-road vehicle crashes.

The group of juniors accounted for 19,216 total emergencies caused by an off-road vehicle crash. Overall, 8,770 of these emergencies occurred during the 25 weeks preceding their birthday and 10,072 occurred during the 25 weeks following their birthday (Fig 1). The 1,302 excess crashes was equivalent to a relative risk increase of 15% following a birthday (95% confidence interval 12% to 18%). The absolute increase in risk was equal to an average of about 2.7 additional crashes per 1000 juniors each week following a birthday (18.3 vs 21.0, p < 0.0001). Analyses restricted to the two months before and after each birthday yielded a relative risk increase of 7% following a birthday (95% confidence interval 1% to 12%). The group of juveniles accounted for the remaining 15,218 emergencies and showed no significant increase in crashes following a birthday (19.2 vs. 19.8, p = 0.31).

**Fig 2:**
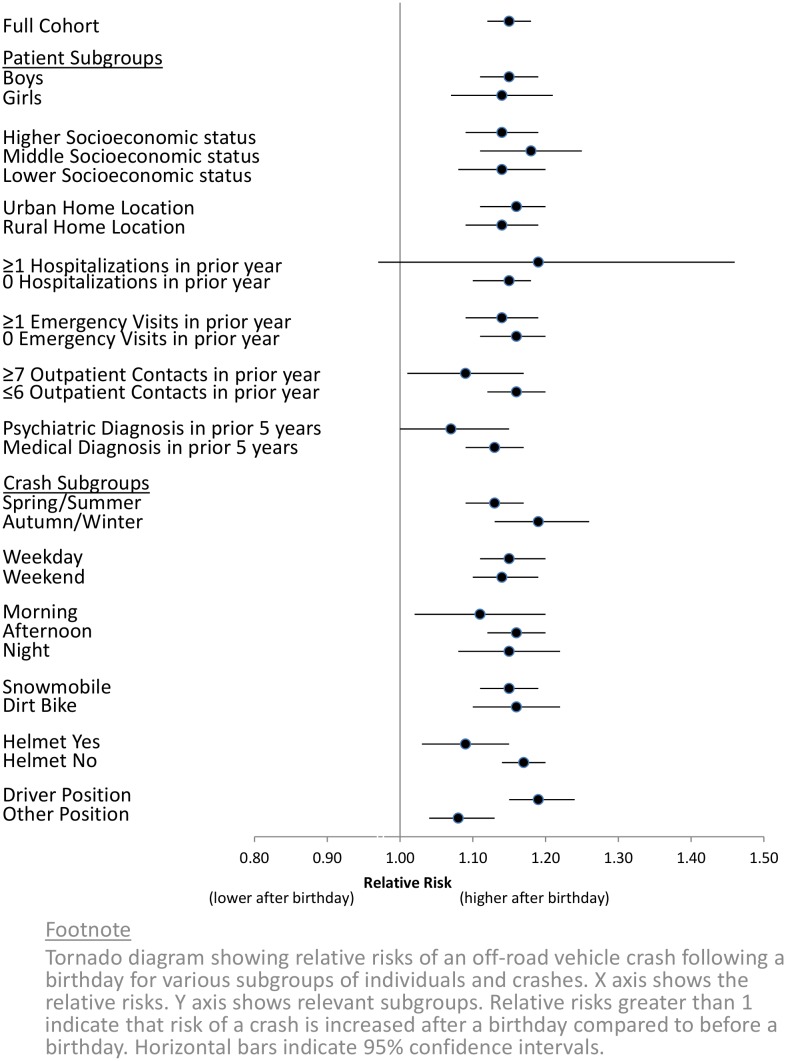
Relative risks for Juniors in different subgroups.

**Fig 3:**
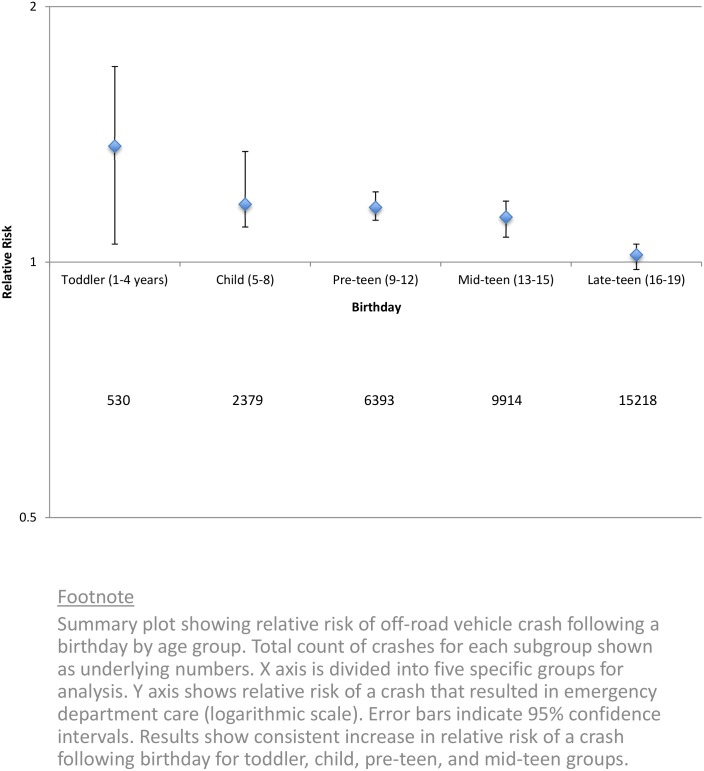
Relative risk of a crash by age group.

The increased risk among juniors was significant for different ages, sexes, times of the day, seating positions and vehicle types. Crashes were more prevalent among juniors with a higher socio-economic status, yet the relative risk of a crash following a birthday extended from lower to higher levels of socio-economic status (Fig 2). General measures of past medical history were not significant predictors of the relative risk of crash following a birthday. The increase in risk following a birthday was accentuated for drivers and attenuated for those who wore helmets. All age groups among juniors showed a significant relative increase in risk (Fig 3) and the 14th birthday showed the highest increase in absolute risk (9.7 additional crashes per week)

**Fig 4:**
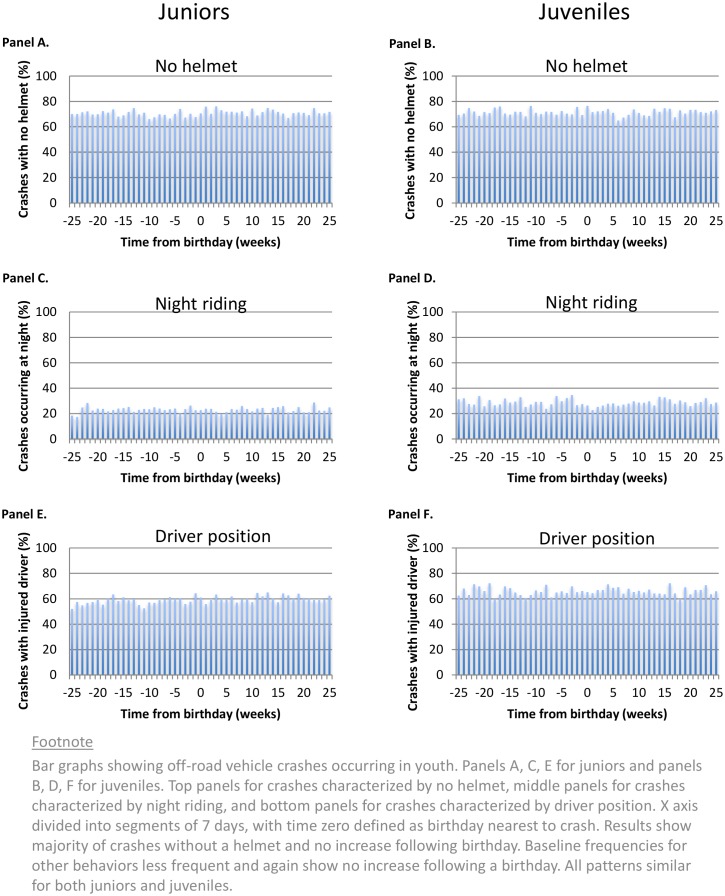
Indicators of safety behavior in injured youth.

Birthdays were not associated with a sudden increase in maturity as assessed by the measured safety behaviors of youth involved in a crash. About two-thirds were not wearing a helmet–and this proportion was no different for youth involved in a crash before or after a birthday (Fig 4). Similarly, the proportion driving at night was no different for youth involved in a crash before or after a birthday. The lack of growth in safe driving behaviors was equally evident for both juniors and juveniles involved in a crash. Secondary analyses of specific birthdays showed no significant exceptions to this overall pattern.

We directed special attention to seating position under the assumption that parental permissions linked to birthdays primarily focus on a youth being a driver rather than passenger (or being a passenger rather than a bystander). As expected, most juniors were passengers rather than drivers. Juniors, however, were somewhat more likely to be drivers during the weeks following their birthday than the weeks before their birthday (odds-ratio = 1.10, 95% confidence interval 1.04–1.17). Injuries were significantly more extensive for drivers than passengers across all measures of crash severity (S2 Table). The increased crash risk after a birthday, however, was still significant when tested for the subgroup of youth involved as passengers in a crash.

Of the 35,202 total emergency department visits, 49% (n = 17,341) received a higher triage urgency score, 7% (n = 2,637) led to hospital admission, and a total of 3,739 hospital bed-days were accounted for over the study interval. The crashes attributed to a birthday equated to about 1 extra hospital admission per month in Ontario. Among youth admitted to hospital, 47% (n = 1,273) required surgery and 6% (n = 165) required mechanical ventilation. At discharge, 91% (n = 2,367) returned home, 8% (n = 212) were transferred to long-term care, and 1% (n = 18) were dead. Measures of injury severity were not statistically significantly different before and after a birthday (and similar for juniors and juveniles).

## Interpretation

We studied a sample of over thirty thousand youth to explore the association of birthdays with the subsequent risk of an off-road vehicle crash. We observed more than one thousand extra emergency department visits caused by crashes among juniors in the six months following their birthday compared to the six months before their birthday, equivalent to about a 15% relative risk increase. The absolute increase in risk was greatest with the 14th birthday. The relative increase in risk was not observed for juvenile birthdays, perhaps explained by the start of road driving (and decreased off-road activities) [55]. These data suggest that birthdays are associated with an increase in subsequent emergencies for off-road vehicle crashes in juniors.

Several limitations of our research merit attention. We have no data about crashes that did not receive emergency medical care, including those that resulted in vehicle damage only, minor injury, or immediate death [64]. We also lack data about alcohol [69], drugs [70], peer pressure [71], parental supervision [72], and other factors that influence risky behavior [73–74]. Our study assessed individuals according to first presentation and thereby underestimated total rates of recurrence. Our study was based in Canada, which has a higher frequency of snowmobile crashes relative to other industrial countries [47,75–77]. Our study did not control for the distribution of birthdays throughout the year as a potential confounder and there remain opportunities for future research. Our study cannot easily separate the effects of a birthday from the effects of growing older; however, the data show no further increases between the fifth and twenty-fifth week following a birthday (Fig 1).

A further limitation of our research is that the observed relationship between birthdays and crashes may be difficult to modify. Parents may fail to read or follow scientific research recommendations, underestimate the adverse consequences of a crash [78–79], or believe that aggregate statistics do not apply to their individual children [80–81]. Policies attempting to influence rider behavior might also be perceived as threats to privacy, autonomy, and liberty [82–84]. In addition, excessive restrictions might deter youth from engaging in other outdoor activities that promote physical fitness, an appreciation of nature, and overall quality of life [85–88]. Some increase in injuries might also be explained as natural acts of childhood rebellion despite effective parenting and community supports [89–90].

The complete mechanism underlying the observed rise in crashes among youth is unknown [91–92]. Parents may bestow privileges by granting the freedom to ride after an age milestone, such as the youth’s fourteenth birthday. Attractive miniature off-road vehicles designed specifically for juniors age 6 years are often marketed as gifts for special occasions [93–94]. Prevailing community and cultural norms shape the practices of parents [95]. marketing behaviors of retailers [96], and expectations of youth [97–99]. Privileges like hunting, driving, drinking, flying and voting all have age thresholds when freedoms are granted; thus, youths may have similar expectations about riding an off-road vehicle [55,100–101].

Our study is the first to observe a link between childhood birthdays and medical emergencies, hence justifying the need for more research. The overall rates of emergencies were consistent with past literature, but our analysis excluded off-road vehicle injuries treated by primary care physicians outside emergency medical settings and those that resulted in immediate death [102]. We observed a helmet compliance rate that agrees with current literature although far below legal standards [103]. Measures of injury severity including triage scores [104], length of hospital stay, and discharge status were consistent with the literature yet lack details on long-term outcomes for survivors [105–106]. Similar to most preceding studies, the typical injured youth was a young boy in a rural area [107–108].

Our study demonstrates the low compliance rate of helmets among seriously injured youth despite laws requiring riders to wear a helmet [49]. One explanation might be misunderstandings around the risk of operating an off-road vehicle that lead to undervaluing the importance of safety [109–112]. In addition, youth are usually financially dependent upon their parents, so that the failure to wear a helmet could also reflect more general challenges inside the home [113–114]. Policies that required a helmet to be included with the sale of every vehicle might help address this gap, although might neglect passengers who did not purchase an off-road vehicle [115–116]. Furthermore, subsidized programs for exchanging damaged or undersized helmets might ensure sustained helmet use [117].

Off-road vehicles enhance the quality of life for youth in rural regions yet require careful riding during every trip. Parents and their children should consider the benefits of wearing a helmet [118], emergency preparedness, and other safety strategies before each ride [119–120]. The results of this study could inform parental gifting habits, commercial marketing policies, and public awareness campaigns [121–122]. More efforts encouraging riders to wear helmets also merit reinforcement by primary care physicians [123–125]. Our study also shows significant differences in injury severity according to seating position, suggesting that past experience as a passenger does not necessarily prepare youth to drive safely. Age milestones provide a convenient time to grant riding privileges to youth, yet adventures will sometimes lead to misadventures.

## Ethics Statement

The study protocol was approved by the Research Ethics Board of the Sunnybrook Health Sciences Centre. The views expressed are those of the authors and do not necessarily reflect the Institute for Clinical Evaluative Sciences or the Ontario Ministry of Health and Long-term care.

## Supporting Information

S1 Fig Relative risks in different subgroups of juveniles. (JPG)

S1 Table More characteristics of injured youth. (PDF)

S2 Table More crash characteristics. (PDF)

S3 Table Seating position and crash severity in all patients. (PDF)

## References

[1] The World Health Organization. WHO global status report on road safety 2013: supporting a decade of action. World Health Organization, 2013.

[2] Novak JA, Hafner JW, Aldag JC, Getz MA. Evaluation of a standardized all-terrain vehicle safety education intervention for youth in rural Central Illinois. J Prim Care Community Health. 2013 1;4(1):8–13. 10.1177/2150131912446374 23799684

[3] US Government Accountability Office. All-terrain vehicles: how they are used, crashes, and sales of adult-sized vehicles for children’s use. Publication No. GAO-10-418. April 2010. Accessed on May 31 2014 available at http://www.gao.gov/products/GAO-10-418

[4] World Health Organization. International statistical classification of diseases and related health problems. Geneva (Switzerland): World Health Organization; 2010.

[5] Rodgers GB, Adler P. Risk factors for all-terrain vehicle injuries: a national case-control study. Am J Epidemiol. 2001 6 1;153(11):1112–8. 10.1093/aje/153.11.1112 11390331

[6] Shulruf B, Balemi A. Risk and preventive factors for fatalities in All-terrain Vehicle Accidents in New Zealand. Accid Anal Prev. 2010 3;42(2):612–8. 10.1016/j.aap.2009.10.007 20159086

[7] Lord S, Tator CH, Wells S. Examining Ontario deaths due to all-terrain vehicles, and targets for prevention. Can J Neurol Sci. 2010 5;37(3):343–9. 10.1017/S0317167100010234 20481268

[8] Pelletier JS, McKee J, Ozegovic D, Widder S. Retrospective review of all-terrain vehicle accidents in Alberta. Can J Surg. 2012 8;55(4):249–53. 10.1503/cjs.036210 22617540 PMC3404145

[9] Rice MR, Alvanos L, Kenney B. Snowmobile injuries and deaths in children: a review of national injury data and state legislation. Pediatrics. 2000 3;105(3 Pt 1):615–9. 10699118 10.1542/peds.105.3.615

[10] Rowe B, Milner R, Johnson C, Bota G. Snowmobile-related deaths in Ontario: a 5-year review. CMAJ. 1992 1 15;146(2):147–52. 1735039 PMC1488372

[11] Specialty Vehicle Institute of America. State all-terrain vehicle requirements. Irvine, CA: Government Relations Office, Specialty Vehicle Institute of America; 2 2012 Accessed on January 26, 2016 available at: http://www.svia.org/Downloads/SVIA-Summary-Chart-February-2012.pdf

[12] Keenan HT, Bratton SL. All-terrain vehicle legislation for children: a comparison of a state with and a state without a helmet law. Pediatrics. 2004 4;113(4):e330–4. 15060263 10.1542/peds.113.4.e330

[13] Weiss H, Agimi Y, Steiner C. Youth motorcycle-related brain injury by state helmet law type: United States, 2005–2007. Pediatrics. 2010 12;126(6):1149–55. 10.1542/peds.2010-0902. Epub 2010 Nov 15. 21078726

[14] Adams LE, Aitken ME, Mullins SH, Miller BK, Graham J. Barriers and facilitators to all-terrain vehicle helmet use. J Trauma Acute Care Surg. 2013 10;75(4 Suppl 3):S296–300. 10.1097/TA.0b013e318292421f 23702632

[15] Denning GM, Jennissen CA, Harland KK, Ellis DG, Buresh CT. Off-highway vehicle parks: combining environment, knowledge, and enforcement for all-terrain vehicle injury prevention. Accid Anal Prev. 2013 3;52:64–70. 10.1016/j.aap.2012.12.015 23298708

[16] McBride AS, Cline DM, Neiberg RH, Westmoreland KD. Pediatric all-terrain vehicle injuries: does legislation make a dent? Pediatr Emerg Care. 2011 2;27(2):97–101. 10.1097/PEC.0b013e31820942f8 21252816

[17] Bell DL, Breland DJ, Ott MA. Adolescent and young adult male health: a review. Pediatrics. 2013 9;132(3):535–46. 10.1542/peds.2012-3414 23940241

[18] Patton GC, Coffey C, Cappa C, Currie D, Riley L, Gore F, et al Health of the world's adolescents: a synthesis of internationally comparable data. Lancet. 2012 4 28;379(9826):1665–75. 10.1016/S0140-6736(12)60203-7 22538181

[19] Viner RM, Coffey C, Mathers C, Bloem P, Costello A, Santelli J, et al 50-year mortality trends in children and young people: a study of 50 low-income, middle-income, and high-income countries. Lancet. 2011 4 2;377(9772):1162–74. 10.1016/S0140-6736(11)60106-2 21450338

[20] Borse NN, Gilchrist J, Dellinger AM, Rudd RA, Ballesteros MF, Sleet DA. CDC Childhood Injury Report: Patterns of Unintentional Injuries among 0–19 Year Olds in the United States, 2000–2006. Atlanta (GA): Centers for Disease Control and Prevention, National Center for Injury Prevention and Control; 2008 10.1097/01.FCH.0000347986.44810.59

[21] Dunlop SM, Romer D. Adolescent and young adult crash risk: sensation seeking, substance use propensity and substance use behaviors. J Adolesc Health. 2010 1;46(1):90–2. 10.1016/j.jadohealth.2009.06.005 20123263

[22] Bell CC, McBride DF. Affect regulation and prevention of risky behaviors. JAMA. 2010 8 4;304(5):565–6. 10.1001/jama.2010.1058 20682937

[23] Clarke S, Robertson IT. A meta-analytic review of the big five personality factors and accident involvement in occupational and non-occupational settings. J Occup Organ Psychol. 2005;78(3):355–376. 10.1348/096317905X26183

[24] Ouimet MC, Brown TG, Guo F, Klauer SG, Simons-Morton BG, Fang Y, et al Higher Crash and Near-Crash Rates in Teenaged Drivers With Lower Cortisol Response: An 18-Month Longitudinal, Naturalistic Study. JAMA Pediatr. 2014 4;168(6):517–22. 10.1001/jamapediatrics.2013.5387 24710522 PMC4139916

[25] Jonah BA. Sensation seeking and risky driving: a review and synthesis of the literature. Accid Anal Prev. 1997 9;29(5):651–65. 10.1016/S0001-4575(97)00017-1 9316713

[26] Kendler KS, Ohlsson H, Sundquist K, Sundquist J. Peer deviance, parental divorce, and genetic risk in the prediction of drug abuse in a nationwide Swedish sample: evidence of environment-environment and gene-environment interaction. JAMA Psychiatry. 2014 4;71(4):439–45. 10.1001/jamapsychiatry.2013.4166 24576925 PMC4002385

[27] Li K, Simons-Morton BG, Vaca FE, Hingson R. Association between riding with an impaired driver and driving while impaired. Pediatrics. 2014 4;133(4):620–6. 10.1542/peds.2013-2786 24639277 PMC3966504

[28] Landen MG, Bauer U, Kohn M. Inadequate supervision as a cause of injury deaths among young children in Alaska and Louisiana. Pediatrics. 2003 2;111(2):328–31. 10.1542/peds.111.2.328 12563059

[29] Duke NN, Pettingell SL, McMorris BJ, Borowsky IW. Adolescent violence perpetration: associations with multiple types of adverse childhood experiences. Pediatrics. 2010 4;125(4):e778–86. 10.1542/peds.2009-0597 20231180

[30] National Safe Kids Campaign. Follow the Leader: A National Study of Safety Role Modeling Among Parents and Children. 2005. Accessed June 1 2014, available: http://www.safekids.org/assets/docs/ourwork/research/research-report-safe-kids-week-2005.pdf

[31] Gentile DA, Li D, Khoo A, Prot S, Anderson CA. Mediators and Moderators of Long-term Effects of Violent Video Games on Aggressive Behavior: Practice, Thinking, and Action. JAMA Pediatr. 2014 5 1;168(5):450–7. 10.1001/jamapediatrics.2014.63 24663396

[32] Christakis DA, Garrison MM, Herrenkohl T, Haggerty K, Rivara FP, Zhou C, et al Modifying media content for preschool children: a randomized controlled trial. Pediatrics. 2013 3;131(3):431–8. 10.1542/peds.2012-1493 23420911 PMC3581844

[33] Strasburger VC; American Academy of Pediatrics. Council on Communications and Media. Policy statement—children, adolescents, substance abuse, and the media. Pediatrics. 2010 10;126(4):791–9. 10.1542/peds.2010-1635 20876181

[34] Council on Communications and Media. American Academy of Pediatrics. Policy statement—sexuality, contraception, and the media. Pediatrics. 2010 9;126(3):576–82. 10.1542/peds.2010-1544 20805150

[35] Dunlop SM, Romer D. Associations between adolescent seatbelt non-use, normative perceptions and screen media exposure: results from a national US survey. Inj Prev. 2010 10;16(5):315–20. 10.1136/ip.2009.025999 20805616

[36] Agence France-Presse. "Police Charge Justin Bieber with Assault." n.p. Globalpost, 30 Jan. 2014. Web. Accessed on June 1 2014 available: http://www.globalpost.com/dispatch/news/afp/140130/police-charge-justin-bieber-assault

[37] Shults RA, West BA, Rudd RA, Helmkamp JC. All-terrain vehicle-related nonfatal injuries among young riders in the United States, 2001–2010. Pediatrics. 2013 8;132(2):282–9. 10.1542/peds.2013-0751 23821703 PMC5751408

[38] Larson AN, McIntosh AL. The epidemiology of injury in ATV and motocross sports. Med Sport Sci. 2012;58:158–72. 10.1159/000338728 22824845

[39] Fonseca AH, Ochsner MG, Bromberg WJ, Gantt D. All-terrain vehicle injuries: are they dangerous? A 6-year experience at a level I trauma center after legislative regulations expired. Am Surg. 2005 11;71(11):937–40. 16372612

[40] Dolan MA, Knapp JF, Andres J. Three-wheel and four-wheel all-terrain vehicle injuries in children. Pediatrics. 1989 10;84(4):694–8. 2780132

[41] Masten SV, Foss RD, Marshall SW. Graduated driver licensing and fatal crashes involving 16- to 19-year-old drivers. JAMA. 2011 9 14;306(10):1098–103. 10.1001/jama.2011.1277 21917580

[42] Rutledge PC, Park A, Sher KJ. 21st birthday drinking: extremely extreme. J Consult Clin Psychol. 2008 6;76(3):511–6. 10.1037/0022-006X.76.3.511 18540744 PMC2668868

[43] Lewis MA, Neighbors C, Lee CM, Oster-Aaland L. 21st birthday celebratory drinking: evaluation of a personalized normative feedback card intervention. Psychol Addict Behav. 2008 6;22(2):176–85. 10.1037/0893-164X.22.2.176 18540715 PMC2758637

[44] Iosua EE, Gray AR, McGee R, Landhuis CE, Keane R, Hancox RJ. Employment Among Schoolchildren and Its Associations With Adult Substance Use, Psychological Well-being, and Academic Achievement. J Adolesc Health. 2014 10; 55(4):542–8. 10.1016/j.jadohealth.2014.03.018 24861950

[45] Schoenhals M, Tienda M, Schneider B. The educational and personal consequences of adolescent employment. Social Forces, 1998;77(2):723–761. 10.2307/3005545

[46] Statistics Canada. Estimates of population, by age group and sex for July 1, Canada, provinces and territories, annual (persons). CANSIM (database) Table 051–0001. Accessed on June 6, 2014 available at http://www.statcan.gc.ca/tables-tableaux/sum-som/l01/cst01/demo02a-eng.htm

[47] Statistics Canada. Land and freshwater area, by province and territory. Natural Resources Canada. Accessed on August 24, 2014 available at http://www.statcan.gc.ca/tables-tableaux/sum-som/l01/cst01/phys01-eng.htm

[48] Ontario road safety annual report. Toronto: Ontario Ministry of Transportation, 2008. Accessed on June 6, 2014 available at: http://www.mto.gov.on.ca/english/safety/orsar/orsar08/index.shtml

[49] Criminal Code. R.S.C. 1985, c. C-46 s. 253. Accessed on June 6, 2014 available at: http://laws-lois.justice.gc.ca/eng/acts/C-46/page-123.html

[50] Ontario Medical Association. Position paper: All-terrain vehicles (ATVs) and children’s safety. August, 2009. Accessed on June 6, 2014 available at: https://www.oma.org/Resources/Documents/All-Terrainvehicles.pdf

[51] Off-Road Vehicles Act. R.S.O. 1990, c. 0.4 ss. 4(1), 4(2), 19(1). Accessed on June 6, 2014 available at: http://www.e-laws.gov.on.ca/html/statutes/english/elaws_statutes_90o04_e.htm

[52] Sessions SY, Detsky AS. Washington, Ottawa, and health care reform: a tale of 2 capitals. JAMA. 2010;303(20):2078–9. 10.1001/jama.2010.654 20501930

[53] Iron K, Goel V, Williams JI. Concordance with hospital discharge abstracts and physician claims for surgical procedures in Ontario. North York (ON): Institute for Clinical Evaluative Sciences; 1995.

[54] Williams JL, Young W. Inventory of studies on the accuracy of Canadian health administrative databases. Ottawa (ON): Institute for Clinical Evaluative Sciences; 1996.

[55] Macpherson AK, Schull M, Manuel D, Cernat G, Redelmeier DA, Laupacis A. Injuries in Ontario. ICES atlas. Toronto (ON): Institute for Clinical Evaluative Sciences; 2005.

[56] Highway Traffic Act. R.S.O. 1990 c. H.8 ss. 37. Accessed on June 20, 2014 available at: http://www.elaws.gov.on.ca/html/statutes/english/elaws_statutes_90h08_e.htm

[57] Canadian Institute for Health Information. CIHI data quality study of emergency department visits for 2004–2005. Vol. 2: Main study findings Ottawa: CIHI, 2008.

[58] Iron K, Zagorski BM, Sykora K. Living and dying in Ontario: an opportunity for improved health information ICES investigative report. Toronto (ON): Institute for Clinical Evaluative Sciences; 2008.

[59] Wilkins R. Use of postal codes and addresses in the analysis of health data. Health Rep. 1993;5(2):157–77. 8292756

[60] Wilkins R. Automated Geographic Coding Based on the Statistics Canada Postal Code Conversion Files, Including Postal Codes to December 2003. Ottawa, Canada: Health Analysis and Measurement Group, Statistics Canada; 2004.

[61] Glazier RH, Creatore MI, Agha MM, Steele LS; Inner City Toronto Time Trends Working Group. Socioeconomic misclassification in Ontario's Health Care Registry. Can J Public Health. 2003;94(2):140–3. 12675172 10.1007/BF03404588 PMC6988580

[62] Williams JI, Young W. A Summary of Studies on the Quality of Health Care Administrative Databases in Canada In: Goel V, Williams JI, Anderson GM, Blackstien-Hirsch P, Fooks C et al, editors. Patterns of Health Care in Ontario. The ICES Practice Atlas. Ottawa (ON): Canadian Medical Association; 1996.

[63] Juurlink DN, Preyra C, Croxford R, Chong A, Austin P, Tu J, et al Canadian Institute for Health Information Discharge Abstract Database: A Validation Study. Toronto (ON): Institute for Clinical Evaluative Sciences; 2006.

[64] Wijeysundera DN, Beattie WS, Austin PC, Hux JE, Laupacis A. Epidural anaesthesia and survival after intermediate-to-high risk non-cardiac surgery: a population-based cohort study. Lancet. 2008;372(9638):562–9. 10.1016/S0140-6736(08)61121-6 18692893

[65] Gershon AS, Warner L, Cascagnette P, Victor JC, To T. Lifetime risk of developing chronic obstructive pulmonary disease: a longitudinal population study. Lancet. 2011;378(9795):991–6. 10.1016/S0140-6736(11)60990-2 21907862

[66] Redelmeier DA. The exposure-crossover design is a new method for studying sustained changes in recurrent events. J Clin Epidemiol. 2013 9;66(9):955–63. 10.1016/j.jclinepi.2013.05.003 23850556

[67] Redelmeier DA, May SC, Thiruchelvam D, Barrett JF. Pregnancy and the risk of a traffic crash. CMAJ. 2014 7;186(10):742–50. 10.1503/cmaj.131650 24821870 PMC4081196

[68] McNemar Q. Note on the sampling error of the difference between correlated proportions or percentages. Psychometrika 1947;12:153–7. 10.1007/BF02295996 20254758

[69] Whitehill JM, Rivara FP, Moreno MA. Marijuana-Using Drivers, Alcohol-Using Drivers, and Their Passengers: Prevalence and Risk Factors Among Underage College Students. JAMA Pediatr. 2014 7;168(7):618–24. 10.1001/jamapediatrics.2013.5300 24820649 PMC4090688

[70] Asbridge M, Hayden JA, Cartwright JL. Acute cannabis consumption and motor vehicle collision risk: systematic review of observational studies and meta-analysis. BMJ. 2012 2 9;344:e536 10.1136/bmj.e536 22323502 PMC3277079

[71] Santos RG, Durksen A, Rabbanni R, Chanoine JP, Lamboo Miln A, Mayer T, et al Effectiveness of peer-based healthy living lesson plans on anthropometric measures and physical activity in elementary school students: a cluster randomized trial. JAMA Pediatr. 2014 4;168(4):330–7. 10.1001/jamapediatrics.2013.3688 24515353

[72] Bell DL, Breland DJ, Ott MA. Adolescent and young adult male health: a review. Pediatrics. 2013 9;132(3):535–46. 10.1542/peds.2012-3414 23940241

[73] Lee RD, Fang X, Luo F. The impact of parental incarceration on the physical and mental health of young adults. Pediatrics. 2013 4;131(4):e1188–95. 10.1542/peds.2012-0627 23509174 PMC3608482

[74] Gardner HG; American Academy of Pediatrics Committee on Injury, Violence, and Poison Prevention. Office-based counseling for unintentional injury prevention. Pediatrics. 2007 1;119(1):202–6. 10.1542/peds.2006-2899 17200289

[75] The World Health Organization. WHO global status report on road safety 2015: supporting a decade of action. World Health Organization, 2015.

[76] Rice MR, Alvanos L, Kenney B. Snowmobile injuries and deaths in children: a review of national injury data and state legislation. Pediatrics. 2000 3;105(3 Pt 1):615–9. 10699118 10.1542/peds.105.3.615

[77] Pierz JJ. Snowmobile injuries in North America. Clin Orthop Relat Res. 2003 4;(409):29–36. Review. 10.1097/01.blo.0000057781.10364.c9 12671482

[78] Kahneman D, Tversky A. On the psychology of prediction. Psychol Rev 1973; 80:237–51. 10.1037/h0034747

[79] Slovic P. Perception of risk. Science. 1987 4 17; 236(4799):280–85. 10.1126/science.3563507 3563507

[80] Osterberg L, Blaschke T. Adherence to medication. N Engl J Med. 2005 8 4;353(5):487–97. Review. 10.1056/NEJMra050100 16079372

[81] Diekema DS. Improving childhood vaccination rates. N Engl J Med. 2012 2 2;366(5):391–3. 10.1056/NEJMp1113008 22296072

[82] Hayek FA. Law, legislation and liberty: a new statement of the liberal principles of justice and political economy. Abingdon (OX): Routledge: 2012.

[83] Kass NE. An ethics framework for public health. Am J Public Health. 2001 11;91(11):1776–82. 10.2105/AJPH.91.11.1776 11684600 PMC1446875

[84] Hooper C, Spicer J. Liberty or death; don't tread on me. J Med Ethics. 2012 6;38(6):338–41. 10.1136/medethics-2011-100085 22398414

[85] Thompson Coon J, Boddy K, Stein K, Whear R, Barton J, Depledge MH. Does participating in physical activity in outdoor natural environments have a greater effect on physical and mental wellbeing than physical activity indoors? A systematic review. Environ Sci Technol. 2011 3 1;45(5):1761–72. 10.1021/es102947t 21291246

[86] Mann MJ, Leahy JE. Connections: Integrated Meanings of ATV riding among club members in Maine. Leisure Sciences. 2009 9 1:31(4):384–96. 10.1080/01490400902988317

[87] Ferreira I, van der Horst K, Wendel-Vos W, Kremers S, van Lenthe FJ, Brug J. Environmental correlates of physical activity in youth—a review and update. Obes Rev. 2007 3;8(2):129–54. 10.1111/j.1467-789X.2006.00264.x 17300279

[88] Whitaker RC. Obesity prevention in pediatric primary care: four behaviors to target. Arch Pediatr Adolesc Med. 2003 8;157(8):725–7. 10.1001/archpedi.157.8.725 12912775

[89] Ter Bogt TF, Keijsers L, Meeus WH. Early adolescent music preferences and minor delinquency. Pediatrics. 2013 2;131(2):e380–9. 10.1542/peds.2012-0708 23296443

[90] Zahrt DM, Melzer-Lange MD. Aggressive behavior in children and adolescents. Pediatr Rev. 2011 8;32(8):325–32. 10.1542/pir.32-8-325 21807873

[91] Grimes DA, Schulz KF. Bias and causal associations in observational research. Lancet. 2002;359(9302):248–52. 10.1016/S0140-6736(02)07451-2 11812579

[92] Karhausen LR. Causation in epidemiology: a Socratic dialogue: Plato. Int J Epidemiol. 2001;30(4):704–6. 10.1093/ije/30.4.704 11511586

[93] Hafner JW, Getz MA, Begley B. All-terrain vehicle dealership point-of-sale child safety compliance in Illinois. Pediatr Emerg Care. 2012 8;28(8):739–44. 10.1097/PEC.0b013e31826249f9 22858746

[94] All-terrain vehicle injury prevention: two-, three-, and four-wheeled unlicensed motor vehicles. Pediatrics. 2000 6;105(6):1352–4. 10835081 10.1542/peds.105.6.1352

[95] Njoroge WF, Elenbaas LM, Garrison MM, Myaing M, Christakis DA. Parental cultural attitudes and beliefs regarding young children and television. JAMA Pediatr. 2013 8 1;167(8):739–45. 10.1001/jamapediatrics.2013.75 23778788

[96] Deyton L, Sharfstein J, Hamburg M. Tobacco product regulation—a public health approach. N Engl J Med. 2010 5 13;362(19):1753–6. 10.1056/NEJMp1004152 20410498

[97] Bansal V, Fortlage D, Lee J, Kuncir E, Potenza B, Coimbra R. A 21-year history of all-terrain vehicle injuries: has anything changed? Am J Surg. 2008 6;195(6):789–92. 10.1016/j.amjsurg.2007.05.049 18367134

[98] Scutchfield SB. All-terrain vehicles: injuries and prevention. Clin Orthop Relat Res. 2003 4;(409):61–72. 10.1097/01.blo.0000060441.40507.3e 12671486

[99] Ellis S, Gauvain M. Children’s Development Within Social Context. Hillsday (NJ): Lawrence Erlbaum Associates: 2013.

[100] Highway Traffic Act. R.S.O. 1990, c. H.8 ss. 37(1). Accessed on June 6, 2014 available at: http://www.e-laws.gov.on.ca/html/statutes/english/elaws_statutes_90h08_e.htm#BK67

[101] Liquor Licence Act. R.S.O. 1990. c. L.19 ss. 30. Accessed on June 10, 2014 available at: http://www.e-laws.gov.on.ca/html/statutes/english/elaws_statutes_90l19_e.htm#BK34

[102] Collins CL, Smith GA, Comstock RD. Children plus all nonautomobile motorized vehicles (not just all-terrain vehicles) equals injuries. Pediatrics. 2007 7;120(1):134–41. 10.1542/peds.2006-3612 17606570

[103] Gittelman MA, Pomerantz WJ, Groner JI, Smith GA. Pediatric all-terrain vehicle-related injuries in Ohio from 1995 to 2001: using the injury severity score to determine whether helmets are a solution. Pediatrics. 2006 6;117(6):2190–5. 10.1542/peds.2005-2603 16740864

[104] Sandler G, Soundappan SS, Manglick MP, Fahy FE, Ross F, Lam L, et al Pediatric "off-road vehicle" trauma: determinants of injury severity and type. Pediatr Emerg Care. 2012 12;28(12):1328–33. 10.1097/PEC.0b013e318276b0d2 23187993

[105] Killingsworth JB, Tilford JM, Parker JG, Graham JJ, Dick RM, Aitken ME. National hospitalization impact of pediatric all-terrain vehicle injuries. Pediatrics. 2005 3;115(3):e316–21. 10.1542/peds.2004-1585 15741358

[106] Yuma PJ, Maxson RT, Brown D. All-terrain vehicles and children: history, injury burden, and prevention strategies. J Pediatr Health Care. 2006 Jan-Feb;20(1):67–70. 10.1016/j.pedhc.2005.10.002 16399484

[107] Krauss EM, Dyer DM, Laupland KB, Buckley R. Ten years of all-terrain vehicle injury, mortality, and healthcare costs. J Trauma. 2010 12;69(6):1338–43. 10.1097/TA.0b013e3181fc5e7b 21150516

[108] Brown RL, Koepplinger ME, Mehlman CT, Gittelman M, Garcia VF. All-terrain vehicle and bicycle crashes in children: epidemiology and comparison of injury severity. J Pediatr Surg. 2002 3;37(3):375–80. 10.1053/jpsu.2002.30826 11877651

[109] Robertson DW, Lang BD, Schaefer JM. Parental attitudes and behaviours concerning helmet use in childhood activities: Rural focus group interviews. Accid Anal Prev. 2014 9;70:314–9. 10.1016/j.aap.2014.04.011 24836477

[110] Ranney ML, Mello MJ, Baird JB, Chai PR, Clark MA. Correlates of motorcycle helmet use among recent graduates of a motorcycle training course. Accid Anal Prev. 2010 11;42(6):2057–62. 10.1016/j.aap.2010.06.017 20728662

[111] Finnoff JT, Laskowski ER, Altman KL, Diehl NN. Barriers to bicycle helmet use. Pediatrics. 2001 7;108(1):E4 10.1542/peds.108.1.e4 . 11433083

[112] Rutter DR, Quine L, Albery IP. Perceptions of risk in motorcyclists: unrealistic optimism, relative realism and predictions of behaviour. Br J Psychol. 1998 11;89 (Pt 4):681–96. 10.1111/j.2044-8295.1998.tb02710.x 9854808

[113] Adams LE, Aitken ME, Mullins SH, Miller BK, Graham J. Barriers and facilitators to all-terrain vehicle helmet use. J Trauma Acute Care Surg. 2013 10;75(4 Suppl 3):S296–300. 10.1097/TA.0b013e318292421f 23702632

[114] Khambalia A, MacArthur C, Parkin PC. Peer and adult companion helmet use is associated with bicycle helmet use by children. Pediatrics. 2005 10;116(4):939–42. 10.1542/peds.2005-0518 16199705

[115] Aitken ME, Graham CJ, Killingsworth JB, Mullins SH, Parnell DN, Dick RM. All-terrain vehicle injury in children: strategies for prevention. Inj Prev. 2004 10;10(5):303–7. 10.1136/ip.2003.004176 15470012 PMC1730140

[116] Wu BC, Oakes JM. A randomized controlled trial of sport helmet interventions in a pediatric emergency department. Pediatr Emerg Care. 2005 11;21(11):730–5. 16280946 10.1097/01.pec.0000186426.69517.24

[117] Owen R, Kendrick D, Mulvaney C, Coleman T, Royal S. Non-legislative interventions for the promotion of cycle helmet wearing by children. Cochrane Database Syst Rev. 2011 11 9;(11):CD003985 10.1002/14651858.CD003985.pub3 22071810 PMC7390328

[118] Novak JA, Hafner JW, Aldag JC, Getz MA. Evaluation of a standardized all-terrain vehicle safety education intervention for youth in rural Central Illinois. J Prim Care Community Health. 2013 1;4(1):8–13. 10.1177/2150131912446374 23799684

[119] Williams RS, Graham J, Helmkamp JC, Dick R, Thompson T, Aitken ME. A trial of an all-terrain vehicle safety education video in a community-based hunter education program. J Rural Health. 2011 Summer;27(3):255–62. 10.1111/j.1748-0361.2010.00327.x 21729152

[120] Blecker N, Rhee P, Judkins DG, Wynne JL, Friese RS, Kulvatunyou N, et al Pediatric all-terrain vehicle trauma: the epidemic continues unabated. Pediatr Emerg Care. 2012 5;28(5):443–7. 10.1097/PEC.0b013e3182531d20 22531189

[121] Aitken ME, Graham CJ, Killingsworth JB, Mullins SH, Parnell DN, Dick RM. All-terrain vehicle injury in children: strategies for prevention. Inj Prev. 2004 10;10(5):303–7. 10.1136/ip.2003.004176 15470012 PMC1730140

[122] Brann M, Mullins SH, Miller BK, Eoff S, Graham J, Aitken ME. Making the message meaningful: a qualitative assessment of media promoting all-terrain vehicle safety. Inj Prev. 2012 8;18(4):234–9. 10.1136/injuryprev-2011-040132 22101098 PMC3704218

[123] Yanchar NL. Preventing injuries from all-terrain vehicles. Paediatr Child Health. 2012 11;17(9):513–4. 24179426 10.1093/pch/17.9.513 PMC3496361

[124] Trauma Committee of the Canadian Association of Pediatric Surgeons. Canadian Association of Pediatric Surgeons' position statement on the use of all-terrain vehicles by children and youth. J Pediatr Surg. 2008 5;43(5):938–9. 10.1016/j.jpedsurg.2007.12.044 18485971

[125] Burd R. American Pediatric Surgical Association Trauma Committee position statement on the use of all-terrain vehicles by children and youth. J Pediatr Surg. 2009 8;44(8):1638–9. 10.1016/j.jpedsurg.2009.03.026 19635318

