# Supplementary figures and images for: Off-Road Vehicle Crash Risk during the Six Months after a Birthday
Source: PLoS One. 2016 Oct 3;11(10):e0149536. doi: 10.1371/journal.pone.0149536 (PMC5047483; doi:10.1371/journal.pone.0149536)

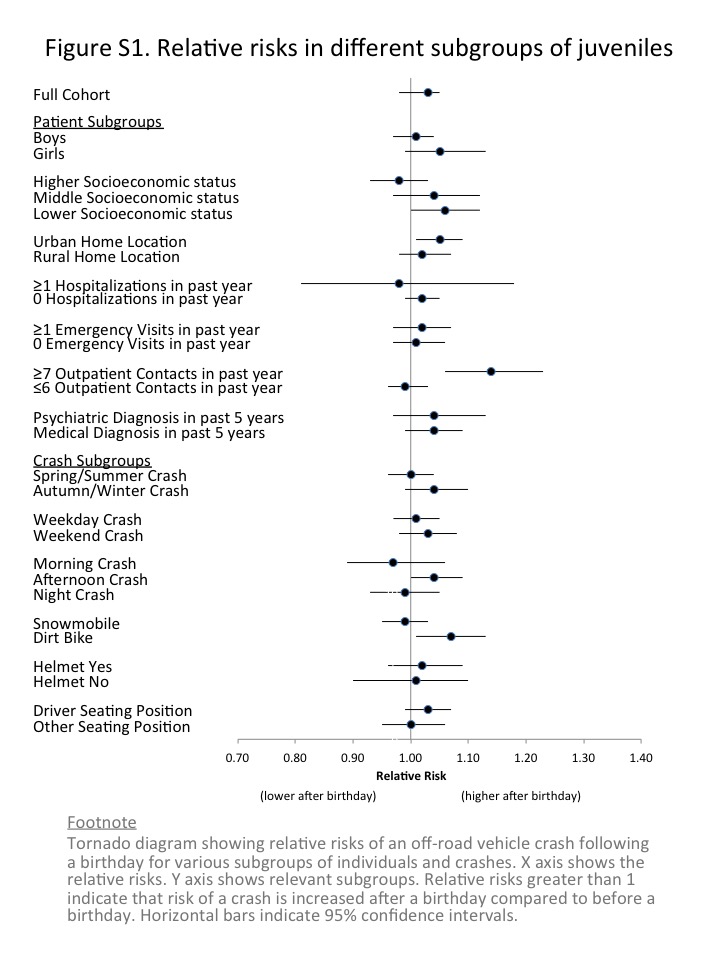

Supplement: S1 Fig — (JPG) [file pone.0149536.s001.jpg]
