# Supplementary material for: Off-Road Vehicle Crash Risk during the Six Months after a Birthday
Source: PLoS One. 2016 Oct 3;11(10):e0149536. doi: 10.1371/journal.pone.0149536 (PMC5047483; doi:10.1371/journal.pone.0149536)
Supplement: S1 Table — (PDF) [file pone.0149536.s002.pdf]

Table S1. More Characteristics of Injured Youth

Characteristic	Juniors (n=20,359)*	Juveniles (n=12,418) †
**Season of birthday**		
Spring	5,249 (26%)	3,460 (28%)
Summer	5,263 (26%)	3,211 (26%)
Autumn	5,094 (25%)	2,875 (23%)
Winter	4,753 (23%)	2,872 (23%)
**Prior diagnoses ¶**		
Concussion (code 850)	657 (3%)	507 (4%)
Behavioral disorder (codes 312, 313, 314)	2,882 (14%)	1,495 (12%)
Learning disorder (code 315)	513 (3%)	130 (1%)
Substance abuse (codes 303, 304, 305)	104 (1%)	427 (3%)
Depression (code 296, 311)	261 (1%)	551 (4%)
Personality disorder (code 301)	75 (0%)	119 (1%)
Epilepsy (code 345)	231 (1%)	140 (1%)
Movement disorder (code 307)	1,034 (5%)	596 (5%)
Asthma (code 493)	4,273 (21%)	1,825 (15%)
Contact dermatitis (code 692)	2,000 (10%)	1,154 (9%)
Otitis media (codes 381, 382)	7,764 (38%)	2,271 (18%)

Footnote

* denotes youth ≤ 15 years of age, defined on day of off-road vehicle crash

† denotes youth ≥ 16 years of age, defined on day of off-road vehicle crash

¶ within 5 years prior to crash

φ All percentages rounded to nearest integer
