# Supplementary material for: Off-Road Vehicle Crash Risk during the Six Months after a Birthday
Source: PLoS One. 2016 Oct 3;11(10):e0149536. doi: 10.1371/journal.pone.0149536 (PMC5047483; doi:10.1371/journal.pone.0149536)
Supplement: S2 Table — (PDF) [file pone.0149536.s003.pdf]

Table S2. More Crash Characteristics

Characteristic	Juniors (n=21,693) *	Juveniles (n=13,509) †
**Vehicle type**		
Snowmobile	15,262 (70%)	9,035 (67%)
Dirt Bike	6,431 (30%)	4,474 (33%)
**Helmet use**		
Yes	6,143 (28%)	3,719 (28%)
No	15,550 (72%)	9,790 (73%)
**Role**		
Driver	12,988 (60%)	8,828 (65%)
Other	8,705 (40%)	4,681 (35%)
**Ambulance arrival**		
Yes	2,319 (11%)	1,866 (14%)
No	19,374 (89%)	11,643 (86%)
**Triage Urgency** §		
High	10,612 (49%)	6,729 (50%)
Low	11,081 (51%)	6,780 (50%)
**Concussion (code 850)**		
Yes	779 (4%)	583 (4%)
No	20,914 (96%)	12,926 (96%)
**Disposition**		
Treated/released	19,775 (91%)	12,258 (91%)
Hospital admission	1,534 (7%)	1,063 (8%)

Footnote

* denotes youth ≤ 15 years of age, defined on day of off-road vehicle crash

† denotes youth ≥ 16 years of age, defined on day of off-road vehicle crash

§ Higher urgency denotes resuscitation, emergency, urgency; lower urgency includes all other triage levels

φ All percentages rounded to nearest integer
