# Supplementary material for: Off-Road Vehicle Crash Risk during the Six Months after a Birthday
Source: PLoS One. 2016 Oct 3;11(10):e0149536. doi: 10.1371/journal.pone.0149536 (PMC5047483; doi:10.1371/journal.pone.0149536)
Supplement: S3 Table — (PDF) [file pone.0149536.s004.pdf]

Table S3. Seating position and crash severity in all patients

	Characteristic	Driver (n=21,816)	Not Driver (n=13,386)	P Value
Full Cohort	**Ambulance arrival**			
	Yes	2863 (13%)	1322 (10%)	<0.001
	No	18953 (87%)	12064 (90%)	
	**Triage urgency †**			
	Higher	11314 (52%)	6027 (45%)	<0.001
	Lower	10478 (48%)	7333 (55%)	
	**Concussion**			
	Yes	907 (4%)	455 (3%)	<0.001
	No	20909 (96%)	12931 (97%)	
	**Transfusion §**			
	Yes	129 (1%)	41 (0%)	<0.001
	No	21687 (99%)	13345 (100%)	
	**Hospital Admission**			
	Yes	1753 (8%)	844 (6%)	<0.001
	No	20063 (92%)	12542 (94%)	
		(n=1,763)	(n=844)	
Admitted patients	**ICU admission***			
	Yes	127 (7%)	38 (4%)	0.007
	No	1626 (93%)	806 (96%)	
	**Discharge status**			
	Alive – home	1583 (90%)	784 (93%)	0.035
	Alive - long-term care	154 (9%)	58 (7%)	
	Dead	16 (1%)	<=5 (0%)	

Footnote

† Higher denotes resuscitation, emergency, urgency; lower denotes all other triage levels

§ includes transfusions of albumin, blood, blood components, plasma, platelets, or red cells.

¶ All percentages rounded to nearest integer

* ICU admission determined by mechanical ventilation
